# Immune cells in the epithelial immune microenvironment of psoriasis: emerging therapeutic targets

**DOI:** 10.3389/fimmu.2023.1340677

**Published:** 2024-01-04

**Authors:** Lisha Li, Jiaye Lu, Jun Liu, Junchao Wu, Xinyue Zhang, Yu Meng, Xiying Wu, Zongguang Tai, Quangang Zhu, Zhongjian Chen

**Affiliations:** ^1^ Shanghai Skin Disease Hospital, School of Medicine, Tongji University, Shanghai, China; ^2^ Shanghai University, School of Medicine, Shanghai, China; ^3^ Shanghai Engineering Research Center of External Chinese Medicine, Shanghai, China

**Keywords:** psoriasis, epithelial microenvironment, inflammatory skin disease, immune cells, cytokines, therapeutic targets

## Abstract

Psoriasis is a chronic autoimmune inflammatory disease characterized by erroneous metabolism of keratinocytes. The development of psoriasis is closely related to abnormal activation and disorders of the immune system. Dysregulated skin protective mechanisms can activate inflammatory pathways within the epithelial immune microenvironment (EIME), leading to the development of autoimmune-related and inflammatory skin diseases. In this review, we initially emphasized the pathogenesis of psoriasis, paying particular attention to the interactions between the abnormal activation of immune cells and the production of cytokines in psoriasis. Subsequently, we delved into the significance of the interactions between EIME and immune cells in the emergence of psoriasis. A thorough understanding of these immune processes is crucial to the development of targeted therapies for psoriasis. Finally, we discussed the potential novel targeted therapies aimed at modulating the EIME in psoriasis. This comprehensive examination sheds light on the intricate underlying immune mechanisms and provides insights into potential therapeutic avenues of immune-mediated inflammatory diseases.

## 1 Introduction

A chronic inflammatory skin condition, psoriasis affects 2-3% of people worldwide (1–4). It encompasses four types, each characterized by distinct pathological features and clinical presentations: chronic plaque, pustular, erythrodermic, and articular psoriasis (5, 6). Among these, chronic plaque psoriasis (commonly referred to as psoriasis vulgaris) is the most prevalent, accounting for approximately 85-90% of all cases (7–9). Psoriasis has a complicated etiology that involves genetic, epigenetic, and environmental variables (10). Over the past two decades, psoriasis treatments have primarily consisted of topical medications (such as corticosteroid creams, retinoid creams, calcineurin inhibitors, keratinocytes (KCs), and antimicrobial agents), oral medications or injections (such as immunosuppressants and biologics), and light therapy (such as ultraviolet B (UVB) irradiation) (11–14). Nonetheless, these treatments offer only temporary relief, while a definitive cure remains elusive (15).

In recent years, the close association between immune-mediated inflammatory skin diseases and epithelial immune microenvironment (EIME) has been under intensive focus (16). Under normal conditions, immune cells should be coordinated, but in psoriasis, their proliferation and differentiation are abnormally regulated, leading to rapid maturation and arrangement of KCs on the skin surface, forming typical scaly plaques (17, 18). These plaques are accompanied by immune dysfunction, including dysregulation of innate and adaptive immune functions, imbalance of immune cell and cytokine interactions in the epidermis, and over proliferation of KCs (19–21). Immune cells and cytokines play an important part in the pathogenesis of psoriasis (22). The therapeutic approaches usually target these immune cells and modulate cytokines to alleviate symptoms and control disease progression (23). Despite some research on psoriasis, there are many unanswered questions, including how the interactions between activated immune cells and cytokines contribute to the pathogenesis of psoriasis. Therefore, an in-depth study and improvement of the correlation between EIME is essential to elucidate the pathogenesis of psoriasis and develop efficient therapies. Throughout this article, we first described the pathogenesis of psoriasis from the perspective of the abnormal activation of immune cells and the release of cytokines. Secondly, the key role of EIME in psoriasis is explained. Subsequently, we analyzed the interactions between a range of innate and adaptive immune cells in inducing the pathogenesis of psoriasis and summarized the inflammatory circuits associated with psoriasis. Finally, potential novel targeted therapies for targeting immune cells and modulating EIME in psoriasis are discussed. In contrast to other psoriasis reviews, this review emphasizes the crucial role of multiple immune cells in the pathogenesis of psoriasis from the perspective of immune cells in EIME and concludes with a systematic review of targeted therapeutic agents against cytokines secreted by immune cells in recent years. In conclusion, this review concentrates on the major skin cell types implicated in the psoriasis pathogenesis and delves into their roles within the inflammatory circuit.

## 2 Pathogenesis of psoriasis

Psoriasis is a chronic inflammatory skin disease whose pathogenesis involves the interaction of genetic and environmental factors (24). Several studies have shown that HLA-Cw6 is one of the most significant disease alleles (25, 26). In addition, some studies have shown that there is a certain association between the HLA-Cw1 gene and some Asian people with high risk of psoriasis (27). The melanocyte derived protein ADAMTSL5 (28) and the Cathelicidin LL37 fragment have also been identified as psoriasis antigens (29). Studies have shown that various keratins such as Keratin 6, Keratin 16 and Keratin 17 are markers of the proliferation of psoriatic KCs. However, only Keratin 17 has been identified as driving keratinocyte hyperproliferation and inflammatory responses (30–32). Previous studies have shown that the environmental factors that aggravate psoriasis are stress, seasonal change, infection, sun exposure, and beta-blocker use. Previous studies have shown that environmental factors that aggravate psoriasis include skin irritation, air pollutants, smoking and drinking habits, infections, sun exposure, drug use and vaccination. In addition, the occurrence of psoriasis is also closely related to whether people are obese, whether they suffer from diabetes, whether their blood lipids are abnormal, whether they suffer from high blood pressure and mental stress (33). In addition, epigenetics is an important factor in the development of psoriasis (34). Epigenetics refers to chemical modifications on genomic DNA, such as DNA methylation and chromatin conformation changes. Recent studies have found that patients with psoriasis have a different pattern of epigenetic modifications than normal individuals, which may lead to aberrant gene expression and disruption of the immune system (35). In particular, differences were observed in epigenetic modifications of certain immune-related genes, which may play a key role in psoriasis susceptibility and progression (36). Moreover, the genetic mechanism of psoriasis is relatively complex and may involve the interaction of multiple genes. Overall, psoriasis is the result of an interaction of genetic and environmental factors. Specifically, genetic factors may make individuals more sensitive to environmental stimuli, thereby increasing the risk of developing the disease. However, genetic and environmental interactions between different people can lead to differences in the condition. In the prevention and treatment of psoriasis, a combination of genetic and environmental factors is essential to better understand and manage the disease.

Psoriasis is a prevalent chronic inflammatory skin condition defined by the hyperproliferation of KCs and immune cell infiltration (10). In clinical practice, managing psoriasis involves modulating the immune system to selectively inhibit immune cell activation, as well as cytokine production, proliferation, and differentiation (37, 38). In previous studies, tumor necrosis factor-alpha (TNF-α) emerged as a pivotal factor in the emergence of chronic inflammatory skin diseases, including systemic lupus erythematosus and psoriasis (39, 40). Initially successful in treating rheumatoid disorders, TNF-α inhibitors were later extended to include the treatment of psoriasis and psoriatic arthritis (PsA) (41, 42). However, the effectiveness of TNF-α inhibitors in psoriasis is based on indirect adaptive immune modulations, especially on the IL-23/IL-17A axis (43). Etanercept, a TNF-α inhibitor, exerts its efficacy through the downregulation of IL-17A, as evident in clinical trials (44), confirming the earlier viewpoint. The Th17/Th1 inflammatory pathway works together in psoriasis through mechanisms that promote immune responses, increase the release of inflammatory factors, and over activate the proliferation of epidermal cells. Th17 and Th1 cells interact with each other in psoriasis. Th17 cells primarily produce pro-inflammatory cytokines such as IL-17 and IL-22, while Th1 cells produce Interferon (IFN) -γ and other cytokines. IL-17 and IL-22 promote the inflammatory response by prompting epidermal cells to produce chemokines that attract other immune cells (e.g., neutrophils) into the skin. At the same time, IFN-γ secreted by Th1 cells also enhances the inflammatory response. The combined effect of IL-17 and IFN-γ exacerbates the hyperproliferation of epidermal keratinocytes, leading to the distinctive scaly lesions of psoriasis. In addition, IL-23 contributes to the differentiation and activation of Th17 cells, while also stimulating the activity of Th1 cells. This further enhances the activation of the Th17/Th1 inflammatory pathway and drives the inflammatory response (20, 45, 46). Typically, the correlation between IL-17A and TNF-α is rather complex, as they synergistically regulate aberrantly expressed keratinocyte genes in various psoriatic lesion sites (47, 48). The regulation of IL-17A and TNF-α engages distinct pathogenetic mechanisms, with IL-17A/IL-23 as the primary pathogenetic axis in psoriasis, while TNF-α assumes a supportive role (49, 50). IL-23 and IL-17A are pivotal inflammatory factors in the pathogenesis of psoriasis, with IL-23 promoting the secretion of IL-17 and IL-21 by Th17 cells, enhancing neutrophil infiltration and inducing inflammation in psoriasis (51, 52).

In addition to the two primary targets, IL-23 and IL-17, several other members of the interleukin family are closely associated with psoriasis, including IL-6, IL-36, IL-12, and IL-22 (53–60). Notably, a positive feedback loop was noted between IL-6 and IL-17, wherein the presence of IL-6 stimulates the secretion of IL-17A and vice versa (61). Moreover, IL-17A triggers the production of other inflammatory factors, such as IL-1β and TNF-α, intensifying the inflammatory responses and exacerbating the inflammatory milieu (62). Additionally, IL-1β and TNF-α play pivotal roles in inflammatory regulation and promote the development of Th17 cells caused by TGF-β and IL-6, further amplifying IL-17 production (63). This amplification contributes significantly to the generation and activation of Th17 cells. Furthermore, IL-6 augments the promotional effect of IL-23 on Th17 cells, resulting in increased IL-17 production and sustained activity of Th17 cells (64, 65). These intricate interactions synergistically modulate immune responses within the inflammatory environment, bearing crucial implications on the pathogenesis and progression of inflammatory diseases. Generalized pustular psoriasis (GPP) is usually characterized by abnormal activation of IL-36. IL-38, also belongs to the IL-36 subfamily and exerts its anti-inflammatory properties by preventing the release of pro-inflammatory cytokines (66). A recent study has unveiled the involvement of IL-9 in psoriasis development through the activation of Janus kinase-signal transducer and activator of transcription (JAK-STAT), phosphoinositide 3-kinase (PI3K), and Mitogen-Activated Protein Kinase (MAPK) signaling pathways (67). Furthermore, IL-12 primarily acts on STAT3, promoting the production of IL-17A, IL-17F, or IL-22 while stabilizing Th17 cells (68, 69). In brief, the pathogenesis of psoriasis encompasses intricate interactions among numerous immune factors and cell types. This interaction drives the chronic inflammatory state of psoriasis.

## 3 Significance of epithelial immune microenvironment in psoriasis

The epidermis, dermis, and subcutaneous tissue make up the three separate layers of the skin, the biggest organ in the body (70). The epidermis is the outermost barrier, primarily consisting of KCs, melanocytes, T lymphocytes, and Langerhans cells (LCs) (71). Each of these cell types plays a significant role within the various epidermal layers. Diverse immune cells, including dendritic cells (DCs), CD4^+^ T helper cells(Th), and γδ T cells, reside in the transitional region connecting the epidermis to the dermis (72). Furthermore, macrophages, neutrophils, mast cells, fibroblasts, and neural associated cell types were also observed (73). This intricate mix of cells adds complexity to the skin’s immune system. Autoimmune responses may occur when the skin’s immune system mistakenly targets self-antigens, destroying host cells (71). As the primary defense line of the immune system, the skin’s immune microenvironment is crucial for the emergence of inflammatory diseases.

**Figure 1:**
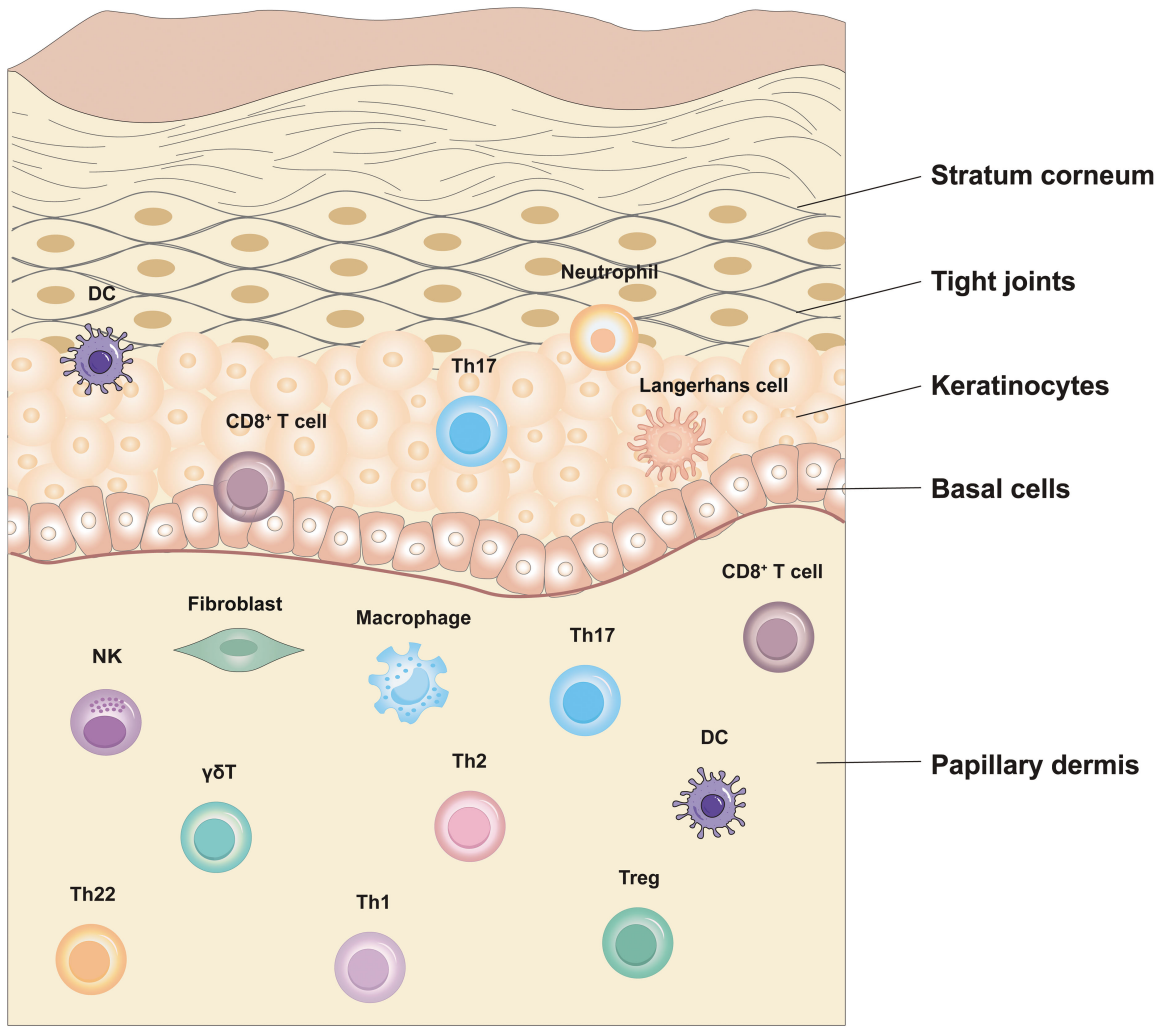
Epithelial immune microenvironment in Psoriasis.

EIME is a crucial element of the skin’s immune response that is situated within the uppermost layer of the epidermis, a mere 0.1-0.2 mm from the skin’s surface. This specialized microenvironment consists of immune cells, cytokines, capillaries and sensory nerve endings, etc (70). In the case of external threats or abnormal conditions, EIME promptly initiates responses and orchestrates the activities of immune cells, ensuring a rapid and effective immune reaction. In psoriasis-associated autoimmune diseases, the main components of the epithelial immune microenvironmental organization: the epidermis and the dermis are the main sites of inflammatory events associated with psoriasis. The epidermis comprises multiple layers of KCs, which form the outermost defense barrier and release inflammatory factors that activate KCs. This amplifies the inflammatory response in autoimmune diseases such as psoriasis. Immune cells within skin tissues secrete cytokines that work in a coordinated manner to mount an effective immune response and collaborate with inflammatory factors to maintain immune homeostasis. The predominant immune cell types found in the epidermis include LCs and CD8^+^ T cells. Meanwhile, the dermis houses an abundance of macrophages, DCs, and fibroblasts. Among them, CD8^+^ T cells rapidly release inflammatory factors after recognizing the antigenic load delivered by antigen-presenting cells (such as DCs). These cytokines, in turn, facilitate the recruitment of lymphocytes, DC maturation, and the recruitment and activation of NK cells (**Figure 1**).

The core characteristic of the immune response in psoriasis is the abnormal activation and interaction of immune cells (74). The immunological processes of this disease are triggered by the presence of abnormal antigens: either self-antigens or exogenous antigens (75, 76). KCs in psoriasis patients are innate immune cells and can be triggered by various factors, leading to a stress response (15, 77, 78). During this process, KCs release nucleotides and antimicrobial peptides, promoting the maturation of DCs (79, 80). This allows for the capture and presentation of aberrant antigens (e.g., autoantigens), activating the immune response, particularly the differentiation of Th1 and Th17 cells. These cells promote skin inflammation and the release of pro-inflammatory mediators, such as TNF-α and IL-17, in psoriatic plaques (81, 82). Additionally, macrophages influence the recruitment and activation of neutrophils, which in turn influences the activation and differentiation of T cells (83). T cells are central components of the immune system and are divided into CD4^+^ T cells and CD8^+^ T cells (84). These subsets have various roles in immune responses, including coordination and execution. CD4^+^ T cells, particularly the Th1, Th17, and Th22 subsets, play a significant role in psoriasis (85). After maturation, they accumulate in the epidermis, releasing cytokines, chemokines, and vascular endothelial growth factors, further promoting excessive proliferation of KCs and the release of pro-inflammatory chemical mediators (78, 80, 86). CD8^+^ T cells are also implicated in the pathogenesis of psoriasis (87). Once activated, they can target KCs, leading to cell lysis and worsening skin lesions (88). In summary, the immune response in psoriasis is a highly complex process involving abnormal interactions and activation of various immune cell types. These interactions are the hallmark of psoriasis among dysfunctional cells and provide clues for potential therapeutic targets. Therefore, an in-depth investigation of the intricate mechanisms involving immune cells in the pathogenesis and inflammation of psoriasis from the perspective of EIME is essential.

## 4 Inflammatory role of immune cells in psoriasis

**Figure 2:**
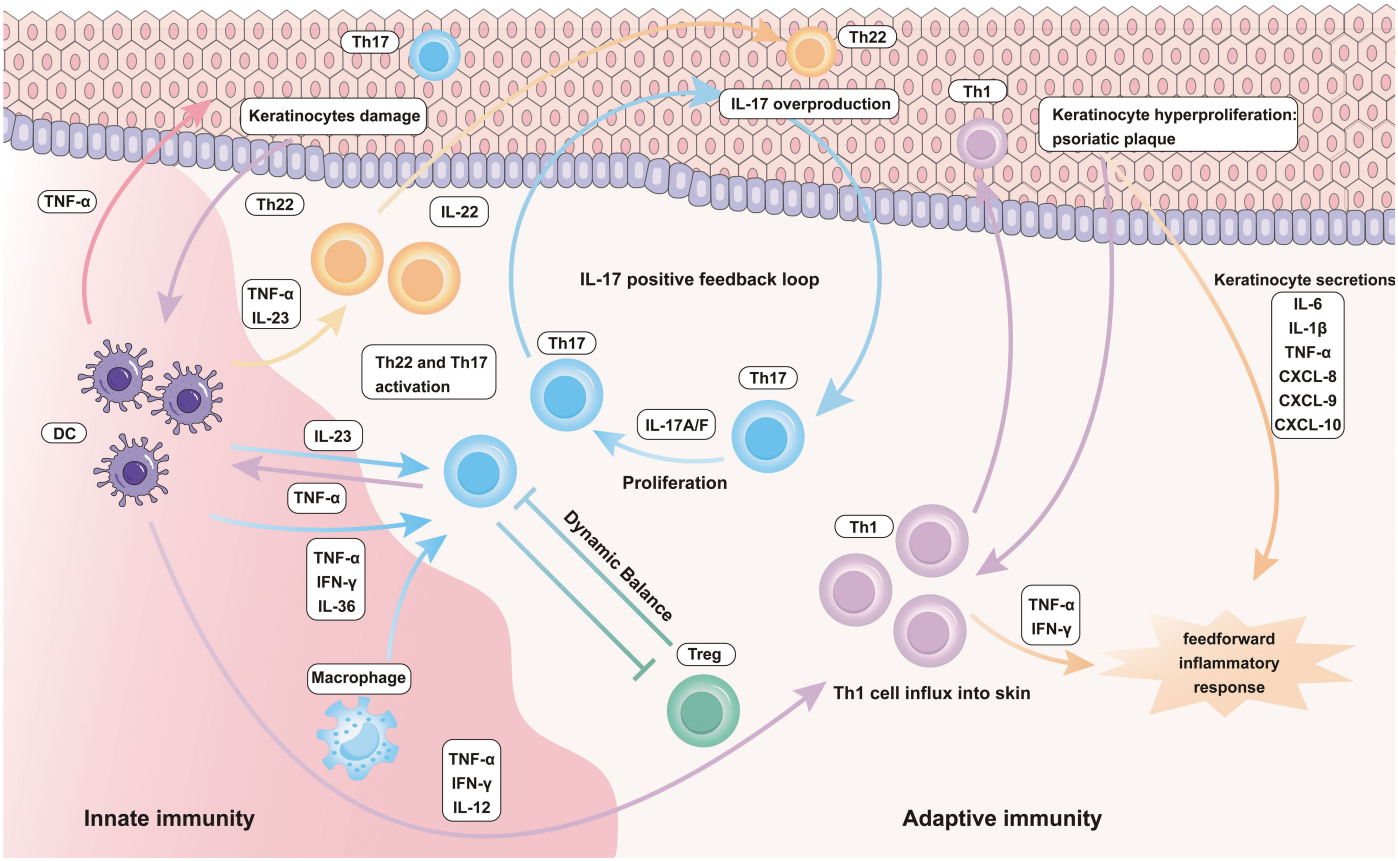
Inflammatory role of innate and adaptive immune cells in Psoriasis.

In autoimmune-mediated skin diseases such as psoriasis, KCs, DCs, macrophages, neutrophils, and T cells assume a pivotal role (80, 89). KCs are primarily situated in the outermost layer of the epidermis, forming the stratum corneum, a fundamental component of the epidermis (90) (91). They represent the innate immune constituents within the skin’s immune system, actively combating external pathogens and engaging in immune and inflammatory responses (18, 92). DCs capture external antigens, presenting them to T cells, activating immune responses, maintaining immune homeostasis, and regulating inflammatory reactions (93, 94). Macrophages engage in the phagocytosis and digestion of pathogens, removal of cellular debris, and the secretion of both pro- and anti-inflammatory signaling molecules, actively participating in immune and inflammatory processes (73, 95). Neutrophils primarily circulate in the blood and have the capacity to migrate to the skin, especially during inflammation (96–98). They are vital components of the innate immune system, responsible for phagocytosis, pathogen destruction, and the clearance of cellular debris from the sites of infection and inflammation. Moreover, T cells are versatile participants in various immune responses within the skin, with a specific emphasis on immune mediated skin disorders, such as psoriasis, where they play a central role in the abnormal activation and immune responses of T cells (99, 100). These diverse cell types collaborate harmoniously within the skin, constituting the skin’s immune system, ensuring an effective response to external threats and pathogens while preserving the delicate balance required for skin health. In situations of inflammation within EIME, these components intricately interact, leading to the establishment of an inflammatory environment in the skin. Consequently, this contributes to the development of chronic inflammatory skin conditions (**Figure 2**).

### 4.1 Innate immune cells in psoriasis

#### 4.1.1 DCs

DCs are often referred to as the sentinels of the immune system because their primary task is to monitor foreign pathogens within the body. They do so by utilizing pattern recognition receptors, such as Toll-like receptors (TLRs), to identify pathogens, which can detect molecular patterns of pathogens. Once DCs detect potential threats, they initiate the immune response process (101). Dermal CD1c^+^CD11b^+^ cDC2s migrate to the surrounding skin epidermis in the early stage of psoriasis, causing rapid inflammation (102).DCs bridge the innate and adaptive immune systems. Activated DCs up-regulate co-stimulatory molecules and generate cytokines, which activate T cells and induce their differentiation into effector cells for carrying out specific immune functions. This mainly encompasses the activation of CD4+ T and CD8+ T cells. In psoriasis, The interaction of DCs with CD4+ T cells significantly affects the differentiation of T cell subtypes such as Th1, Th17 and Th22 (103). These T cell subsets are intricately linked to the inflammation and tissue damage observed in psoriasis. DCs can influence the behavior of these T cell subsets during psoriasis pathology.

Moreover, the clinical development of numerous monoclonal antibodies targeting IL-23 underscores the significance of IL-23 as a pivotal therapeutic target. DCs are major producers of IL-23 (104), possess surface receptors for IL-23 (IL-23R), and are capable of autocrine signaling for IL-23 (105–107). Upregulation of the IL-23 receptor by certain SNP alleles may enhance STAT signaling and thus promote Th17 differentiation (108). The mononuclear cells in peripheral blood from humans or mice, abundant mRNA expression of IL-23p19 makes them the primary source of IL-23 production (104). Additionally, the overexpression of IL-23 has been observed in DC subsets within psoriatic plaques, suggesting that it may play a significant role in the formation and maintenance of psoriatic lesions (109). Furthermore, IL-23 fusion protein induces the production of IL-12 by CD8+ T cells and their subsets. In such cases, DCs secreting IL-12 upregulate the expression of IL-23p19 (110), while inhibiting IL-23p19 in DCs reduces the secretion of TNF-α. On the other hand, supplementation of exogenous IL-23 promotes the secretion of TNF-α (111), indicating an interaction between IL-23/IL-12 and IL-23/TNF-α within DCs. In pustular psoriasis (PP), the activation of TLR7 in DCs triggers a neutrophil response, leading to the excessive secretion of pro-inflammatory factors, such as TNF-α, further accelerating the disease-related deterioration (112).

IFNs may trigger immune dysregulation in autoimmune diseases. DNA/Antimicrobial Peptide LL-37 complexes bind to TLR7, TLR9 in plasmacytoid DCs (pDCs), and TLR8 in bone marrow DCs, promoting the production of IFN-α and IFN-β (113). Additionally, DCs promote immune cell infiltration and activation by secreting IL-15 and IFN-γ (114, 115). The levels of these cytokines have a positive correlation with the severity of psoriasis. Despite the pro-inflammatory role played by DCs in the pathogenesis of psoriasis, some DC subsets may also inhibit the immune response by enhancing regulatory T cell (Treg) production and function (116). In summary, DCs critically contribute to psoriasis pathogenesis by regulating immune activity through antigen presentation, cytokine secretion, and T cells interactions. Furthermore, they participate in psoriasis’ pro-inflammatory processes and potentially inhibit immune responses. Further research will offer deeper insights into the regulatory mechanisms of DCs in psoriasis pathogenesis, potentially unveiling novel targets for disease treatment and intervention.

#### 4.1.2 Macrophages

There are usually two distinct subpopulations of macrophages: M1 macrophages (pro-inflammatory) and M2 macrophages (anti-inflammatory and immunomodulatory), both of which have important regulatory roles in the body’s own immune system and can repair damage associated with inflammation (117).Macrophages play a pivotal role in immune responses from monocyte precursors (118). They release CXCL1, CXCL2, and IL-1 to stimulate and draw neutrophils to the inflammatory site. Subsequently, neutrophils release Recombinant Cathepsin G (CTSG), human neutrophil peptide1-3 (HNP1-3), and Proteinase 3 (PR3), further recruiting monocytes and promoting the inflammatory response (119). In addition, it has been suggested that increased expression of chemokines CXCL9, CXCL10, and CXCL11, which are mainly secreted by monocytes and other cells, has become one of the characteristics of psoriasis and may be candidate markers for psoriasis therapies; however, there is still insufficient evidence to support this idea, and further validation is needed (6, 120). Macrophages polarize into two distinct subtypes: M1 and M2. M1 polarization is mediated by STAT1 and IL-12B, while M2 polarization is mediated by STAT6 and PPAR-γ (121). In psoriasis patients, a higher ratio of M1 to M2 macrophages could be reduced using TNF-α inhibitors, such as adalimumab (122). Furthermore, granulocyte-macrophage colony-stimulating factor (GM-CSF) prolongs the lifespan of neutrophils, further enhancing the inflammatory response of M1 macrophages (123, 124). TLRs, especially TLRs 7-9, are pivotal in activating macrophages and DCs and are implicated in psoriasis pathogenesis. Inhibiting the interaction between TLRs 7-9 and M1 macrophage polarization can reduce inflammation in psoriasis (125). Additionally, macrophages express macrophage-inducible C-type lectin (Mincle), a pattern recognition receptor primarily used to identify damage-associated molecular patterns (DAMPs) and pathogen-associated molecular patterns (PAMPs). In a mouse model of psoriasis, Mincle-expressing macrophages promote the development of psoriatic skin inflammation (126). The macrophages participate in the immune response of psoriasis through bidirectional regulation mechanisms. These molecules impact the activation and differentiation of T cells, particularly Th17 cells, which constitute the predominant inflammatory cell type in psoriatic plaques. Moreover, macrophages produce and release a variety of inflammatory mediators, such as TNF-α, IL-1β, IL-6, IL-12, IFN-γ, and IL-23, which directly or indirectly promote the formation of psoriatic lesions (127–129). Similarly, IL-23 secreted by macrophages promotes Th17 differentiation (108). On the other hand, macrophages can engulf abnormally proliferating KCs, alleviate psoriatic lesions (130), and play multiple roles in the immunopathological process of psoriasis. Further studies would contribute to a deep understanding of their regulatory mechanisms, offering new strategies and targets for treating psoriasis.

#### 4.1.3 Neutrophils

Neutrophils are short-lived granulocytes within the immune system, playing multiple roles in immune responses. These cells also participate in immune reactions through phagocytosis, granule release, reactive oxygen species (ROS) production, and secretion of chemokines and cytokines, and recruit other immune cells, enhancing the host’s immune response (131). In acute inflammation, neutrophils respond rapidly, serving as the body’s initial line of defense in the immune system, reducing the severity of inflammation. However, neutrophils’ function may change in chronic inflammation, leading to tissue damage and excessive immune responses (132). Previous studies have shown that neutrophils play a crucial role in chronic inflammation, including sustained chemotaxis, releasing proteases, and forming neutrophil extracellular traps (NETs) while activating other immune cells. In different stages of psoriasis, neutrophils play critical roles. Interestingly, in the initial phases of plaque psoriasis, they infiltrate the dermal region of psoriatic lesions, subsequently migrating to the epidermis and stratum corneum (133, 134). In GPP, the episodic infiltration of neutrophils leads to symptoms, such as pustules, systemic erythema, and desquamation (135). Furthermore, the interaction between neutrophils and platelets in psoriasis may trigger a prethrombotic state. Moreover, in psoriatic skin lesions, the interaction between platelets and neutrophils might lead to the formation of platelet-neutrophil complexes through the binding of P-selectin on platelets and P-selectin glycoprotein ligand-1 (PSGL-1) on neutrophils. This interaction further promotes the interaction between intercellular adhesion molecule-2 (ICAM-2) on platelets and TNF associated activation protein (TRAP) on neutrophils, thereby enhancing the formation of platelet-neutrophil complexes. This mechanism has been confirmed in the imiquimod-induced psoriasis mouse model, wherein antibody blockade of PSGL-1 on neutrophils reduced the formation of platelet-neutrophil complexes and mitigated the severity of skin lesions (136). Subsequently, the formation of these complexes might lead to a stronger aggregation of immune cells in the psoriatic lesion area, exacerbating the chronic inflammatory response in psoriasis.

#### 4.1.4 Myeloid-derived suppressor cells

Additionally, myeloid-derived suppressor cells (MDSCs) are derived from bone marrow and are precursors of DCs, macrophages, and granulocytes. They are a type of immune regulatory cells mainly produced by pathologically-activated neutrophils and monocytes. MDSCs have a role in regulating Th17 and Treg cell function and can suppress immune cell responses. They can modulate T cells in the event of chronic inflammation. Abnormal expansion of MDSCs with impaired immunosuppressive function may lead to excessive release of pro-inflammatory cytokines, such as IL-23, IL-1β, and IL-6, which may further exacerbate the proliferation and differentiation of Th17 cells, a phenomenon that is commonly seen in patients with psoriasis. The accumulation of MDSCs in the skin is more significant compared to healthy individuals, suggesting their potential involvement in the long-term inflammatory process of psoriasis through the modulation of CD4^+^ T cell differentiation, especially promoting Th17 cell differentiation. Inhibitors of MDSCs, such as gusperimus, reduce their accumulation, decreasing Th17 cell infiltration in the spleen and relieving psoriasis symptoms (137–139). In summary, the interactions between neutrophils and cell types such as monocytes, platelets, and MDSCs profoundly influence the disease’s development and pathogenic mechanisms in the immunopathological process of psoriasis.

### 4.2 Adaptive immune cells in psoriasis

#### 4.2.1 CD4 + T cell

KCs serve as the executors of immune functions in the pathogenesis of psoriasis, while immune cells serve as the key driving force behind psoriasis. CD4^+^ T cells drive autoimmune diseases. The development of psoriasis is significantly influenced by the overactivation of CD4^+^ T cells, with Th17 and Th1 cells polarizing as the major pathogenic cells in psoriasis (140). Each T cell subset has a distinctive cytokine profile that contributes to the specific immune feedback in psoriasis patients (46, 141). The major subsets of CD4 T cells include Th1, Th2, Th17, Th22, and Treg cells (142–144).

#### 4.2.2 Th1 and Th17 cells

Th1 cells constituted the first T cell subset involved in psoriasis vulgaris. Recently, a new population of IL-17-producing CD4^+^ Th cells, known as Th17 cells, along with their associated downstream effector molecules, has been found to be elevated in the skin of psoriasis patients (145, 146). Th17 cells are recognized for their role in protecting epithelial and mucosal tissues, providing immunity against extracellular pathogens, and their contribution in the pathogenesis of inflammatory and autoimmune illnesses, such as psoriasis. These cells function synergistically with Th1 cells in the development of psoriasis (147, 148). Serum cytokines, including TNF-α, IFN-γ, IL-2, IL-6, IL-22, and IL-23, are primarily produced by Th1 and Th17 cells and have emerged as potential biomarkers for psoriasis (149). Th17 cells and the powerful cytokine IL-17A produced by these cells play a pivotal role in the pathogenesis of the aberrant immune response in psoriasis (150), with Notch1 signaling implicated in Th17 cell differentiation and function. In the lesional skin areas of psoriasis, immune cells in the epidermal immune microenvironment, particularly Th17 cells, undergo hyperactivation. Activation of Th17 cells results in their release of large amounts of proinflammatory cytokines, such as IL-17 and IL-22, which in turn trigger an inflammatory response. The cytokines produced by the Th17 cells directly or indirectly affect epidermal cells, particularly KCs. Factors such as IL-17 and IL-22 contribute to the over proliferation and differentiation of KCs, leading to the abnormal proliferation of epidermal cells and the formation of scaly lesions characteristic of psoriasis (39, 151–154). IL-17A is a pro-inflammatory cytokine derived from Th17 cells, which contributes to the pathogenesis of various inflammatory disorders. The number of T cells expressing IL-17A correlates with the severity of skin lesions (37). IL-17A triggers the activation of KCs, resulting in neutrophil recruitment and the secretion of chemokines and pro-inflammatory cytokines (155). The cytokines produced concurrently by activated Th17 cells play a significant role in the pathogenesis of autoimmune diseases, such as psoriasis (156). IL-22 controls KC proliferation and differentiation while facilitating immune system and epithelial cell interactions (142). IL-23, also produced by activated Th17 cells, amplifies the cell response, inducing KC proliferation and other hallmark features of psoriasis (157). IL-23 triggers skin inflammation and stimulates Th17/Th1-polarized immune responses (158). Furthermore, the induction of IL-22 and the involvement of the IL-40 family are crucial in the pathogenesis of psoriasis (24, 157, 159). In addition to the crucial role played by autoreactive T cells and cytokine populations, the IL-23/Th17 pathway and the TNFα-IL-23-Th17 axis is a central signaling pathway that plays a pivotal role in T cell-mediated psoriasis (22, 142, 160).

#### 4.2.3 Th2 cells

IL-17E (also known as IL-25) produced by Th17 cells induces allergic responses and activates the Th2 pathway; nonetheless, the Th2 psoriasis inflammatory circuit has an inhibitory effect in the Th17 cell-mediated inflammation model (37). Activated Th2 cells mediate cellular inflammation through synergistic effects with their secreted pro-inflammatory cytokines, IL-1, IL-12, and IFN-γ. The recruitment and activation of CD4^+^, CD8^+^ T cells, Th17 cells, innate lymphoid cells (ILCs), and γδ T cells subsequently trigger KCs in psoriasis humoral immune response and inflammatory response (142). Remarkably, the role of IL-33 in psoriasis should not be underscored. Several studies have pointed out that IL-33 is significantly increased at the lesion site and in the serum of psoriasis patients (161–164). IL-33, originating from keratinocyte secretion, triggers the expression of psoriasis-associated inflammatory factors and stimulates the progression of skin inflammation in psoriasis (165, 166).

#### 4.2.4 Th22 cells

Th22 cells represent a newly discovered CD4 ^+^ T cell subpopulation abundant in human skin and play a crucial role in epidermal wound healing. These cells possess anti-inflammatory, antibacterial, and antiviral activities with the capacity to differentiate into Th1 or Th2 cells under certain circumstances (167). Th22 cells are characterized by the preferential expression of CCR10, CCR6, and CCR4 and produce a repertoire of cytokines, including IL-22, TNF-α, IL-13, and IL-26 but do not secrete IFN-γ, IL-4, or IL-17. This unique cytokine profile, gene expression pattern, and function distinguish among these molecules. Th22 cells are present in the epidermis during inflammatory dermatosis, wherein skin homing T-lymphocytes contribute to skin homeostasis and immunity (168–170). The predominant secretion of cytokine IL-22 by Th22 cells facilitates epithelial innate immune responses. With other cytokines, IL-22 acts on the skin mucosal barrier and activates KCs, leading to their activation, proliferation, and epidermal hyperplasia (170). In addition to Th17 cells, Th22 also produces IL-22. Th22 cells, mainly derived from the Th17 cell lineage (171, 172). The Th22/IL-22 pathway has a pathogenic function in psoriasis, and evidence suggests that Th22 cells are also involved in psoriasis relapse (142, 156). Furthermore, Th22 cells contribute to adaptive and innate immune responses via the production of T cell- and NK cell-promoting factors, such as IL-15 and IL-7 (170).

#### 4.2.5 Treg cells

Tregs maintain immune tolerance by releasing suppressive cytokines, inducing apoptosis, and inhibiting the secretion of cytokines like IL-2. The dysfunction of Tregs, leading to an inability to control autoimmune inflammation, contributes to the development of psoriasis. The primary functional impairment of Treg cells is linked to the excessive proliferation of pathogenic T cells (113). In psoriatic lesions, forkhead box transcription factor P3 (FOXP3)-positive Treg cells can differentiate into highly pro-inflammatory triple-positive IL-17A^+^/Foxp3^+^/CD4^+^ Th17 cells, sustaining the overall inflammatory process (168). In psoriasis, the IL-23/IL-17 axis of inflammation collaborates with Treg dysfunction, resulting in Th17/Treg imbalance (173). The infiltration and overactivation of effector T cells (Th1, Th2, and Th17) disrupt the balance between Treg and effector T cells and upregulate the pro-inflammatory cytokines (174). Under pro-inflammatory conditions, in the presence of cytokine IL-6, the generation of Treg cells is inhibited, and they are converted into Th17 cells (175). In psoriasis patients, dysfunctional Treg cells in peripheral blood exhibit phosphorylation and abnormal activation of STAT3 in response to the activities of IL-6, IL-21, and IL-23 (168, 176, 177). STAT3 plays a critical role in psoriasis-like inflammation (176) and is involved in the downstream signaling of IL-23 (178), further promoting Th17 polarization. Additionally, IL-23 activates the counter-regulatory function of Treg cells through the STAT3 pathway (24, 173).

#### 4.2.6 CD8 + T cells

The infiltration of lymphocytes occurs in the dermal papilla region of the skin, with CD4^+^ T lymphocytes being the predominant cells. Interestingly, the ratio of these CD4^+^ T and CD8^+^ T cells is reversed in the epidermis. This differentiation suggested a central role of CD8^+^ T cells as the primary drivers of the immune response within the skin. The pathogenesis of psoriasis is influenced by dysfunction within specific T cell subsets, leading to the aberrant release of corresponding cytokines, including IFN-γ, TNF-α, IL-23, and the members of the IL-17 family (179). Inflammatory infiltrates are an early occurrence in psoriasis-affected skin, with various inflammatory cells, such as T lymphocytes, macrophages, mast cells, and polymorphonuclear granulocytes, adhering to the most active regions of the lesion. Notably, the ratio of helper to suppressor T cells in the blood of psoriasis patients is elevated, indicating that suppressor T cells are recruited to the areas of skin lesions (73). Psoriasis is characterized as an IL-17-induced inflammatory skin disease, with pathogenic drivers that encompass autoantigen-induced CD8^+^ T cells (180). Another study employing single-cell transcriptomics compared CD8^+^ T cell transcriptome heterogeneity between psoriasis and healthy skin and showed the presence of common CD8^+^ T cell subsets in both psoriasis and healthy skin along with an increased abundance of CD8^+^ T cells in the psoriatic lesions. CD8^+^ T cells produce IL-17 and other inflammatory cytokines at the sites of active psoriasis and in degenerating skin tissues. These CD8^+^ T cells are pivotal in psoriatic lesion formation, persistence, and recurrence. Liu et al. also investigated the pathogenic role of CD8 T cells in psoriasis by single-cell transcriptomics (88).

#### 4.2.7 TRM cells

Tissue-resident memory T cells, also known as TRM cells, have been discovered in a variety of non-lymphoid organs. Among these, CD8^+^ TRM cells exert potent effector functions (87), such as cytotoxicity, cytokine production, sustained expression of the cytotoxic molecule granzyme B, and the secretion of pro-inflammatory cytokines and chemokines (181). Following viral re-infection, these CD8^+^ TRM cells are crucial for attracting other immune cells to the tissues, such as myeloid cells and circulating memory T cells. They also start a localized immune response that not only involves antigen-specific T cells but also attracts innate immune system cells via antigen-specific mechanisms (182). This behavior is linked to autoimmune conditions like psoriasis. TGF-β serves as a negative regulator of CD8^+^ T cell function and plays a key role in limiting the effector function of cutaneous TRM cells (183). Moreover, other cytokines secreted by cutaneous TRM cells, especially IL-15 and IL-7, play a significant role in inflammatory skin diseases (87). In the context of psoriasis, CD8^+^ CD49a^+^ TRM cells originating from psoriatic lesions generate an IL-17 response that promotes localized inflammation in this dermatological condition. These cells also display a preference for producing IFN-γ during inflammatory dermatological conditions like psoriasis. Importantly, they exhibit high cytotoxicity upon stimulation (184).

### 4.3 Inflammatory role of other immune cells in psoriasis

γδ T cells represent a specialized class of T cells that defy easy classification within the traditional boundaries of adaptive and innate immune cells. These cells exhibit some characteristics of adaptive immune cells while displaying properties typical of innate immune cells. This unique hybrid nature makes γδ T cells intriguing contributors to the immune system. Typically, T cell receptors (TCRs) found in most T cells are comprised of two glycoprotein chains, known as αβ T cells. In contrast, γδ T cells, which are less common, possess a TCR composed of one γ chain and one δ chain. These γδ T cells are predominantly found at mucosal and epithelial locations, including the skin and respiratory, digestive, and reproductive systems, bridging the realms of innate and adaptive immunity. Remarkably, even in the absence of TCR connectivity, γδ T cells generate IL-1 in response to stimuli, such as IL-17, IL-23β, or danger signals, endowing them with a significant role in certain infectious and autoimmune diseases (185). γδ T cells function similar to innate-like cells, amplifying the acquired immune response. They promptly respond to foreign pathogens at an early stage and have the capacity to generate cytokines with known pathogenicity in psoriasis, thus initiating the downstream immune responses (168). γδ T cells detected in various epithelial tissues, including the skin, gut, lung, and genital tract, serve as the primary source of IL-22 (156, 186, 187). Recently, γδ T cells have recently been found to produce IL-17 in psoriasis, contributing to its early production and influencing Th17 cell responses (113, 188, 189). Furthermore, depending on their microenvironment, γδ T cells exhibit functions akin to Th1, Th2, Treg, and Th17 cells (37).

The growth factors produced by γδ T cells, such as VEGF, FGF-2, and IGF-1, along with the induction of antimicrobial peptides in KCs, contribute significantly to epithelial protection and wound repair. γδ T cells also secrete IL-10, which fosters the expansion of CD8^+^ T cells, thereby reducing TNF-α secretion. Furthermore, IL-17-producing γδ T cells play a role in recruiting neutrophils and monocytes to inflammatory sites. In contrast to dendritic epidermal γδ T cells and conventional αβ T cells, dermal γδ T cells express IL-23 receptor, CCR6, the transcription factor RORγ T, and various chemokine receptors, most of which are predominantly expressed chemokine receptors in psoriasis (190). The remaining γδ T cells also express IL-23R and AhR and selectively express TLR2, TLR1, and dectin-1. The expression of AhR is crucial for IL-22 production (168). Additionally, the major inflammatory factors, IL-17 and IL-23, produced by these cells can stimulate each other. IL-23 activates the STAT3 pathway (191). IRF-4, a transcription factor, connects the IL-1R and IL-23R pathways, increasing the production of IL-17 by dermal γδ T cells (192). Psoriasis patients tend to produce memory-like γδ T cells in their skin, which exhibit strong adhesion properties and memory cell characteristics that enable rapid responses to secondary stimuli. This phenomenon is one of the primary reasons for recurrent psoriasis outbreaks (193, 194).

## 5 Emerging therapies targeting immune cells in psoriasis

Psoriasis currently has no known cure, and the treatment relies mostly on managing its symptoms. The conventional treatment options encompass various approaches, including corticosteroids, vitamin D analogs, phototherapy, and systemic therapy (195). However, these treatments have some limitations and associated risks. For instance, corticosteroids are the preferred choice for mild psoriasis as they can rapidly reduce skin inflammation. Nevertheless, their prolonged use may result in skin atrophy and adverse effects (196–198). UVB phototherapy, often used in conjunction with systemic medications like methotrexate, is effective in patients with moderate to severe psoriasis, especially those with arthritis or nail involvement (199). The phototherapy requires regular visits to hospitals or clinics, which can be inconvenient and may induce localized inflammation (200). Methotrexate, a reasonably priced and therapeutically effective systemic therapy drug, is widely employed in psoriasis treatment (201, 202). However, it carries the risk of serious adverse effects, such as liver fibrosis and cirrhosis, necessitating close monitoring and management by both patients and physicians (203). The commonality among these treatments is their potential to alleviate the skin symptoms associated with psoriasis. All these approaches entail some inherent risks and potential adverse effects.

Biologics are widely used in clinical practice, yielding significant clinical outcomes. The concurrent research into small molecule drugs is actively progressing, offering new treatment options for patients who may not tolerate or qualify for biologics. With an improved understanding of the immunopathology of psoriasis, substantial progress is being made in the development of drugs that target the underlying mechanisms of the disease (204, 205) (**Tables 1**, **2**). Next, we provide a comprehensive overview of emerging immunotherapies for psoriasis. This includes the role of biologics targeting specific immune cells or cytokines, small molecule therapies, and future directions for psoriasis treatment.

**Table 1:** Biological agents employed in the management of psoriasis.

Target	Name	Application	Country	Status	NCT
IL-17	Netakimab	Psoriatic Arthritis	Russian	Phase 3	NCT03598751
		Psoriasis	Russian	Phase 2	NCT02762994
IL-17	Brodalumab	Psoriasis Vulgaris	Belgium	Phase 4	NCT04306315
		Psoriatic Arthritis	USA	Phase 2	NCT01516957
		Psoriasis Vulgaris	Germany	Phase 4	NCT03331835
IL-17	CJM112, Secukinumab	Chronic Plaque Psoriasis	USA	Phase 1	NCT01828086
IL-17	Ixekizumab	Moderate-To-Severe Psoriasis	USA	Phase 2	NCT01107457
		Psoriasis, Psoriasis Vulgaris	China	Phase 1	NCT03073213
IL-17	Secukinumab	Psoriasis	Austria	Phase 1	NCT01539213
		Psoriasis, Psoriasis Vulgaris	Germany	Phase 2	NCT02483234
		Psoriasis, Psoriasis Vulgaris	UK	Phase 2	NCT02854163
		Moderate to Severe Plaque-type Psoriasis	USA	Phase 3	NCT01365455 NCT01544595
		Psoriatic Arthritis	USA	Phase 3	NCT01892436
IL-23	Guselkumab	Psoriasis of Scalp, Psoriasis	USA	Phase 4	NCT05858632 NCT05004727
IL-23	Mirikizumab	Psoriasis	Canada	Phase 1	NCT01947933
IL-23	Mirikizumab	Plaque Psoriasis	USA	Phase 2	NCT02899988
IL-23	Mirikizumab	Psoriasis	USA	Phase 3	NCT03482011 NCT03556202
IL-23	Risankizumab	Psoriasis	USA	Phase 4	NCT04630652
IL-23	Risankizumab	Psoriasis	USA	Phase 2	NCT05283135
IL-23	Risankizumab	Moderate to severe plaque psoriasis	German	Phase 3	NCT03255382
IL-23	Tildrakizumab	Psoriasis Vulgaris, Psoriasis, Aging, Epigenetic Disorder, Skin Inflammation	USA	Phase 4	NCT04541329 NCT05390515 NCT04271540 NCT05110313
IL-17/IL-23	Secukinumab, Ixekizumab, Brodalumab, Guselkumab, Risankizumab, Tildrakizumab, Bimekizumab	Psoriasis, Psoriasis Vulgaris	Belgium	Phase 4	NCT04340076
TNF-α	Adalimumab	Plaque Psoriasis	China	Phase 3	NCT03316781
TNF-α	Adalimumab	Chronic Plaque Psoriasis	USA	Phase 3	NCT02581345
TNF-α	Adalimumab	Psoriasis	UK	Phase 1	NCT01899755
TNF-α	Adalimumab	Moderate to severe plaque psoriasis	Russian	Phase 3	NCT02762955
TNF-α	Adalimumab	Psoriasis, Sleep Apnea, Obstructive	Canada	Phase 4	NCT01181570
TNF-α	Adalimumab	Psoriasis, Cardiovascular Disease	USA	Phase 4	NCT01866592 NCT03082729
TNF-α	Adalimumab, ABP 501	Plaque Psoriasis	USA	Phase 3	NCT05073315
TNF-α	Certolizumab	Chronic Plaque Psoriasis, Psoriasis	French	Phase 2	NCT00245765 NCT00329303
TNF-α	Etanercept	Psoriasis	USA	Phase 1	NCT00585650
TNF-α	Etanercept	Psoriasis	UK	Phase 4	NCT01971346
TNF-α	Infliximab	Psoriatic Arthritis	China	Phase 4	NCT00432406
TNF-α	Secukinumab (AIN457)	Psoriasis	UK and Ireland	Phase 3	NCT01961609
TNF-α	Tinefcon	Plaque Psoriasis	India	Phase 4	NCT01373567
IL-12/IL-23	Brepocitinib	Moderate To Severe Plaque Psoriasis	USA	Phase 2	NCT02969018
IL-12/IL-23	Deucravacitinib	PsA	USA	Phase 2	NCT03963401
IL-12/IL-23	Ustekinumab	Psoriasis	USA	Phase 4	NCT05270733
		Psoriatic Plaques Refractory	USA	Phase 2	NCT03553823
		Moderate to Severe Psoriasis	UK	Phase 1	NCT01276847
IL-36	Imsidolimab	GPP	USA	Phase 2	NCT03619902
IL-36	Spesolimab	GPP	Japan	Phase 3	NCT05200247
		GPP	China	Phase 3	NCT05239039
		GPP	USA	Phase 2	NCT03782792

**Table 2:** Small molecule targeted drug employed in the management of psoriasis.

Target	Name	Application	Countries	Status	NCT
JAK3	Tofacitinib	Psoriasis	USA	Phase 1	NCT01736696
JAK1 JAK2 JAK3 TYK2	PF06263276	Psoriasis	USA	Phase 1	NCT02193815
JAK	PREJAK	PsA	Argentina	Phase 2	NCT04821206
JAK1 JAK2 JAK3	Peficitinib	Moderate to Severe Plaque Psoriasis	USA	Phase 2	NCT01096862
JAK1	Abrocitinib	Moderate To Severe Plaque Psoriasis	USA	Phase 2	NCT02201524
JAK1 JAK2	Ruxolitinib	Plaque Psoriasis	USA	Phase 2	NCT00820950 NCT00617994 NCT00778700
JAK3	Tofacitinib	PsA	Germany	Phase 2	NCT04062695
JAK3	Tofacitinib	Moderate to Severe Plaque Psoriasis	USA	Phase 2	NCT00678210 NCT01710046
JAK1JAK2	Baricitinib	Moderate to Severe Plaque Psoriasis	USA	Phase 2	NCT01490632
JAK3	Tofacitinib	Chronic Plaque Psoriasis	USA	Phase 2	NCT00678561 NCT01246583 NCT01831466
PDE4	Hemay005	Moderate to Severe Plaque Psoriasis	China	Phase 2	NCT04102241
PDE4	Orismilast	Psoriasis	USA	Phase 2	NCT05190419
JAK1	Upadacitinib (ABT-494)	PsA	USA	Phase 3	NCT03104374 NCT03104400
JAK3	Tofacitinib	Plaque Psoriasis	China	Phase 3	NCT01815424
JAK3	Tofacitinib	PsA	China	Phase 3	NCT03486457
JAK3	Tofacitinib	PsA	Bangladesh	Phase 3	NCT03736161
JAK3	Tofacitinib	Moderate to Severe Plaque Psoriasis	USA	Phase 3	NCT01309737
JAK3	Tofacitinib	Moderate to Severe Plaque Psoriasis	USA	Phase 3	NCT01186744 NCT01163253 NCT01276639 NCT01241591
JAK3	Tofacitinib	Moderate to Severe Plaque Psoriasis and/or PsA	USA	Phase 3	NCT01519089 NCT01882439 NCT01976364 NCT01877668
PDE4	Hemay005	Moderate to Severe Plaque Psoriasis	China	Phase 3	NCT04839328
PDE4	Roflumilast	Chronic Plaque Psoriasis	USA	Phase 3	NCT04211363 NCT04286607 NCT04211389
PDE4	Apremilast	PsA	USA	Phase 3	NCT01925768 NCT01212770 NCT01307423 NCT01212757 NCT01172938
RORγt	VTP-43742	Psoriasis	USA	Phase 1	NCT03724292 NCT02555709
RORγt	GSK2981278	Psoriasis	Germany	Phase 1	NCT03004846 NCT02548052
RORγt	JTE-451	Plaque Psoriasis	Canada	Phase 1	NCT03018509
RORγt	JTE-451	Plaque Psoriasis	USA	Phase 2	NCT03832738
RORγt	Cedirogant(ABBV-157)	Psoriasis	USA	Phase 1	NCT03922607
RORγt	Cedirogant(ABBV-157)	Psoriasis	USA	Phase 2	NCT05044234
A3 Adenosine Receptor	CF101 (IB-MECA)	Plaque Psoriasis	Israel	Phase 2	NCT00428974
A3 Adenosine Receptor	CF101 (IB-MECA)	Plaque Psoriasis	USA and Bulgaria	Phase 2	NCT01265667
A3 Adenosine Receptor	CF101 (IB-MECA)	Plaque Psoriasis	Bosnia and Herzegovina	Phase 3	NCT03168256

### 5.1 TNF-α inhibitor

**Figure 3:**
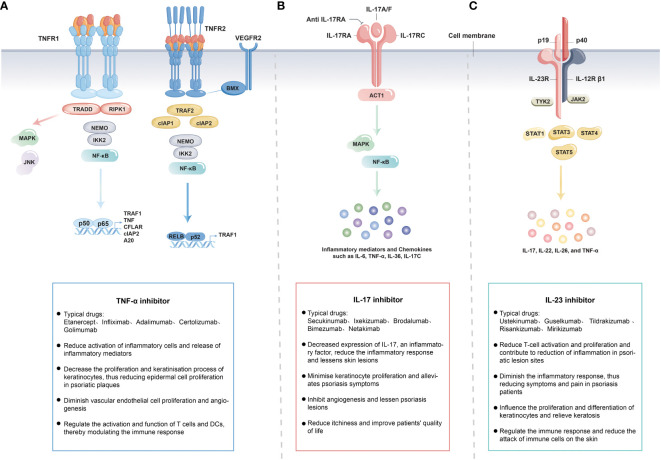
The Mechanisms of TNF-α **(A)**, IL-23 **(B)** and IL-17 **(C)** Inhibitors in Psoriasis Treatment.

TNF receptor 1 (TNFR1) and TNFR2 undergo trimerization, with TNFR1 activating kinases like IκBα-kinase-2 (IKK2) to induce the NF-κB and MAPK pathway (206, 207). This leads to the production of inflammatory factors like IL-17 and TNF, triggering downstream inflammatory responses (208, 209). TNFR2 recruits TNF receptor-associated factor 2 (TRAF2) and cellular inhibitor of apoptosis protein 1 (cIAP1), which, along with TRAF2-cIAP1/2, activate downstream cytokines modifying TNFR1 signaling (such as CFLAR, cIAP2, A20) through the alternative NF-κB pathway (210). TNF exists in both membrane-bound and soluble forms, capable of binding to both TNFR1 and TNFR2. However, the membrane-bound TNF has a higher affinity for TNFR2. In contrast, TNFR2 (also known as p75 or CD120b) has lower affinity for TNF-α but can bind under specific circumstances. (**Figure 3A**). The currently approved and marketed TNF-α inhibitors include Etanercept, Infliximab, Adalimumab, Certolizumab, and Golimumab (211–216). In November 1998, the US the first Food and Drug Administration (FDA) authorized Etanercept, the first anti- tumor necrosis factor inhibitors (TNFi), for the treatment of moderate to severe rheumatoid arthritis (RA) (217). Compared to Etanercept, Infliximab demonstrated rapid and significant efficacy throughout the first stage of therapy (218). Moreover, Infliximab clears skin lesions and PsA patients’ joint discomfort (219). Adults with moderate to severe chronic plaque psoriasis now have an important therapy alternative in Adalimumab, which also offers children and adolescents older than 4 years of age a promising potential systemic treatment option (220). Certolizumab polyethylene glycol (Cimzia) offers pharmacokinetic advantages due to its absence of Fc fragments. It exhibits minimal placental transfer, low relative dose uptake by infants during breastfeeding, and low oral bioavailability (216). Consequently, Certolizumab has emerged as a valuable option for treating moderate to severe plaque psoriasis, providing a therapeutic choice for women of childbearing age. Golimumab is a fully human antibody with a bivalent Fab region that allows it to bind to its soluble and transmembrane forms of the TNF-α protein. Its affinity surpasses that of Infliximab and Adalimumab, effectively reducing the circulating TNF-α protein levels and diminishing its binding to receptors (221). Moreover, Golimumab exhibits lower immunogenicity compared to other TNF-α blockers (222). Nonetheless, patients treated with Adalimumab have reported some adverse events, including thrombocytopenia and leukopenia (223). The FDA approved Golimumab in 2009 for the treatment of PsA and ankylosing spondylitis (AS) (224).

### 5.2 IL-17 inhibitor

IL-17 receptor consists of IL-17RA and IL-17RC, IL-17 binds to the receptor and induces the activation of MAPK pathway and NF-κB pathway, which are prone to produce inflammatory mediators and chemokines, such as IL-6, IL-8, TNF-α, IL-36, IL-17C, in response to IL-17 stimulation (**Figure 3B**). Secukinumab and ixekizumab are antibodies targeting IL-17A (225, 226), whereas brodalumab specifically targets IL-17RA (227). Since IL-17RA chain is involved in several receptor complexes, brodalumab inhibits the actions of various IL-17 variants, including IL-17A, IL-17F, IL-17C, and IL-17E (228, 229). Secukinumab, an innovative subcutaneously administered anti-IL-17 drug, has been approved for use in treating adult patients with active PsA in a number of over 30 countries including the United States, European Union, and Japan. Previous studies have demonstrated the clinical benefits of secukinumab, irrespective of whether patients have previously received treatment with TNFi or other medications like methotrexate (230, 231). Subcutaneous injections of secukinumab exhibited remarkable efficacy across various aspects of PsA and are typically well-tolerated by patients (232). Notably, this efficacy and tolerability can be maintained over the long term, providing patients with a reliable and sustainable treatment option for PsA. Ixekizumab, approved by the US FDA in 2016, is an optimal treatment for plaque psoriasis (233). Ixekizumab has consistently demonstrated impressive efficacy, with no significant differences in the side effects. However, effective clearance usually necessitates ongoing treatment (232). Bimezumab, an antibody that neutralizes IL-17A homodimers and IL-17A-IL-17F heterodimers (234), achieved Psoriasis size and severity index (PASI)-(PASI is a measure of psoriasis lesion area BSA + psoriasis lesion severity, Mild: skin lesions <3% of the area; Moderate: skin lesion area 3%~10% BSA; Severe: skin lesion area >10% BSA.)75 in 93% of patients and PASI-90 in 79% of patients at 12 weeks in phase 2 trials (235). Clinically, both the FDA and European Medicines Agency (EMA) have approved IL-17 inhibitors for the treatment of PsA (230). IL-17 inhibitors may be preferred by patients with extensive skin damage and concurrent PsA or spondylitis (236, 237). These biologics improve psoriasis symptoms by inhibiting the action of IL-17, reducing the activity of Th17 cells, and decreasing the inflammatory response. These therapies have been shown to be clinically effective. In summary, IL-17 inhibitors have consistently demonstrated high effectiveness and safety in psoriasis treatment, providing them with another treatment option, especially for those seeking rapid symptomatic relief.

### 5.3 IL-23 inhibitor

IL-23 consists of p19 and p40 subunits, and the IL-23 receptor consists of IL-12Rβ1 and IL-23R chains. IL-23 binds to the receptor and signals through IL-12Rβ1 and IL-23R, inducing the activation of the JAK-STAT pathway, which can easily produce cytokines, such as IL-17, IL-22, IL-26, and TNF-α (**Figure 3C**). FDA-approved biologic for the direct inhibition of IL-23 was ustekinumab, which functions by binding to the p40 subunit. However, as p40 unit is involved in both IL-23 and IL-12, ustekinumab lacks specificity as an IL-23 inhibitor (238). Subsequently, the FDA approved the use of guselkumab, a specific IL-23 inhibitor, for the treatment of moderate-to-severe psoriasis and PsA (239–241). Moreover, guselkumab binds specifically to the p19 component of IL-23, preventing cytokines from binding to its cell membrane cross-receptor, consequently blocking subsequent intracellular receptor-mediated signaling (208, 242). In a recent cohort study, guselkumab demonstrated superior safety and survival compared to other biologics (including adalimumab, ustekinumab, secukinumab, ixekizumab), highlighting the superiority of guselkumab for the treatment of psoriasis (243). Better long-term efficacy of guselkumab in treating moderate-to-severe psoriasis compared with the use of secukinumab was found in a phase 3 clinical study, which will help healthcare providers make decisions when selecting biologics for the treatment of moderate-to-severe psoriasis (239). Meanwhile, guselkumab remains effective in patients with moderate-to-severe plaque psoriasis who are ineffective on ustekinumab (244). Additionally, in another phase 3 study, guselkumab was found to demonstrate superior efficacy in the treatment of active psoriatic arthritis, with an acceptable benefit-risk profile (242, 245). Two further specialized IL-23 inhibitors, tildrakizumab and risankizumab, have also been developed. In two phase 3 trials, tildrakizumab was found to be highly effective and well tolerated compared to placebo and etanercept in the treatment of patients with moderate to severe chronic plaque psoriasis (246). Subsequent clinical trials have also shown that treatment with tildrakizumab significantly improves the joint and skin manifestations of psoriatic arthritis, with the exception of arthritis of the toes and interphalangeal joints, and is generally well tolerated by patients during the treatment period (247). IL-23 plays a key role in the differentiation and activation of Th17 cells. More comprehensive regulation of the Th17 pathway by simultaneous inhibition of IL-23 reduces inflammation and ameliorates disease (204). Mirikizumab, the fourth anti-IL-23 p19 monoclonal antibody, also showed exceptional effectiveness in treating moderate-to-severe plaque psoriasis (248). In addition to introducing innovative therapeutic options, the creation and success of IL-23 inhibitors have also advanced our knowledge of the etiology of psoriasis. The clinical response rates to these inhibitors have surpassed those of TNF inhibitors (208). In cases where patients had an inadequate response to ustekinumab, switching to a specific IL-23 inhibitor may still be a viable option (244). The superior performance of risankizumab and guselkumab over ustekinumab suggests a protective role of IL-12 in psoriasis (249). Notably, IL-23 inhibition is effective in treating psoriatic skin lesions, but is not efficacious in spondylitis (250). Consequently, IL-23 inhibition is most beneficial for patients with psoriatic skin lesions but without spondylitis. Overall, IL-23 inhibitors have emerged as an effective therapeutic strategy for managing psoriasis. These not only offer patients with treatment options but also elucidate the mechanisms underlying the disease.

### 5.4 IL-36 inhibitor

IL-36 belongs to the important IL-1F family, and it exerts its effects in an autocrine or paracrine manner in several cell types, including KCs (251), epithelial cells (252), and immune cells. The members of the IL-36 subfamily include IL-36α, IL-36β, IL-36γ, and IL-36Rα (253). These three receptor agonists promote inflammation by binding to IL-36R, forming a heterotrimer that alters intracellular structures, thereby activating the MAPK and NF-κB pathways (254). IL-36 may promote pathogenic Th17 responses to enhance inflammation either directly or by inducing IL-23 and acting synergistically with IL-17A (255). Conversely, IL-36Ra serves as a natural antibody effectively inhibiting the initiation and progression of inflammation (254). GPP is primarily characterized by the abnormal activation of IL-36 (256). In individuals with GPP, several gene sites undergo mutations, including CARD14, AP1S3, TNIP1, and SERPINA 3, which are linked to IL-1 and IL-36 signaling pathways, further underscoring the significance of IL-36 in GPP (255, 257). While the mechanism behind IL-36 production during psoriasis development remains unclear, studies have identified an upregulation of IL-36γ expression in serum and skin samples from psoriasis patients, confirming its potential involvement in the initiation and progression of psoriasis (258). Regarding the treatment of GPP, Imsidolimab has displayed promise, but its effectiveness and safety still require substantiation through additional data from phase II and III clinical studies.

### 5.5 JAK inhibitor

Recent studies have revealed that small molecule agents, phosphodiesterase-4 inhibitors and JAK inhibitors, are promising in the treatment of psoriasis. Specifically, phosphodiesterase (PDE) is a crucial molecule involved in the hydrolysis of cyclic adenosine monophosphate (cAMP). The intracellular concentration of cAMP is linked to pro-inflammatory intracellular signaling, making PDE a pivotal molecule in inflammatory diseases such as psoriasis (259). The FDA has approved two PDE-4 inhibitors: the oral formulation of apremilast and the topical cream of roflumilast (260, 261). Several experimental and clinical studies have demonstrated the efficacy and tolerability of these two agents in psoriasis treatment. Apremilast is a small molecule PDE-4 inhibitor lowers cytokine secretion and expression by increasing intracellular cAMP levels (262) and enhances the expression of anti-inflammatory cytokines (263, 264). It also inhibits the activation of NF-κB and MAPK signaling pathways (265). However, PDE-4 inhibitors may have side effects, such as vomiting, which remain a clinical management challenge (266). Roflumilast cream showed excellent efficacy and tolerability in a phase II trial involving psoriasis patients compared to a carrier cream (267). Additionally, butyl 2- (268)benzoate (HFP034) was found to increase cAMP concentration in the skin, inhibit NF-κB activity, and reduce neutrophil infiltration in the skin (269). This finding suggested a potential research avenue for utilizing o-aminobenzoic acid derivatives in psoriasis treatment. Another approach to target the pathogenesis of psoriasis involves JAK inhibitors. JAK1, JAK2, and tyrosine kinase 2(TYK2)are key enzymes implicated in psoriasis, and JAK inhibitors reduce the transcription of pro-inflammatory cytokines by blocking the JAK-STAT pathway (260). Selective TYK2 inhibitor BMS-986165 has shown promising results in phase II clinical data, while PF-06826647 is currently undergoing phase II clinical trials. The TYK2/JAK1 inhibitor Brepocitinib is also in clinical trials for oral and topical treatment (270). In summary, these studies provide a range of options and future research directions for psoriasis treatment, although issues, such as side effects, need to be addressed.

### 5.6 RORγt inhibitor

The RORγT gene plays a significant regulatory function in the development of Th17 cells and has an important regulatory role in synergy with the T-bet transcription factor. This regulatory role can contribute to IFN-γ gene expression and is closely related to the expression level of Th17 cells. VTP-43742, an oral RORγT inhibitor, has demonstrated good efficacy in phase II studies in patients with plaque psoriasis, but has been associated with some adverse effects (271). Currently undergoing a clinical trial in individuals with moderate-to-severe psoriasis, JTE-451 and ABBV-157, novel oral RORγT inhibitor (272), is expected to be a potential treatment option.

### 5.7 TYK2 inhibitor

TYK2 is one of the JAK family genes associated with psoriasis susceptibility genes (18). Functional gene mutations may be associated with abnormalities in multiple cytokine signaling involved in the pathogenesis of psoriasis. Current clinical studies have shown that individuals carrying mutations in the TYK2 gene are not affected by immune mediated inflammatory diseases and are not at increased risk for infections, suggesting that TYK2 inhibitors may be a relatively safe therapeutic target (270, 273). BMS-986165 is a highly selective, orally administered TYK2 inhibitor that blocks STAT1 and IL-23 phosphorylation by inhibiting the activity of both IFN-α and IL-23 (270, 274). PF-06826647 is another TYK2 inhibitor currently in Phase II trials for moderate to severe psoriasis (275).

### 5.8 A3 adenosine receptor

The A3 adenosine receptor, a G protein-coupled receptor, is associated with suppressing inflammatory responses and immune cell activity in previous studies (276, 277). As a result, researchers have begun to explore the use of A3 adenosine receptors as potential targets for psoriasis treatment (278). These agonists inhibit immune cell activity by activating A3 adenosine receptors, thereby reducing inflammation and the proliferation of abnormal skin cells. Although these studies are still in the early stages, CF101 has shown some potential efficacy. One of the advantages of A3 adenosine receptor therapy is that it may have fewer side effects because A3 adenosine receptors are expressed at relatively low levels in other tissues.

### 5.9 mTOR inhibitor

Mammalian target of rapamycin protein (mTOR) is an important protein in a cell signaling pathway that is involved in several biological processes, including cell proliferation, immune regulation, and inflammatory response (279). In patients with psoriasis, the mTOR signaling pathway may be abnormally activated, leading to abnormal proliferation of skin cells and over-activation of the immune system (280, 281). Therefore, researchers have begun to explore mTOR inhibitors as a potential strategy for the treatment of psoriasis. By inhibiting the activity of proteins in the mTOR signaling pathway, the abnormal proliferation of skin cells and overactivation of immune cells can be reduced (282). Some preliminary findings suggest that mTOR inhibitors may have a positive impact on psoriatic lesions, including reduction of skin symptoms and improvement in the appearance of lesions. However, the use of mTOR inhibitors is accompanied by a number of potential side effects and safety concerns (283), so further research is needed to determine the best treatment options and for which psoriasis patients.

## 6 Conclusion and prospects

Regarding autoimmune diseases such as psoriasis, the various immune cell subpopulations associated with the EIME work synergistically to release cytokines, such as TNF-α, IFN-γ, IL-17A, and IL-22, which interact with each other and their secreted inflammatory factors, to create a pro-inflammatory environment within the skin that A dynamic immune homeostasis is maintained. However, at present, the treatment of psoriasis is basically based on cytokines secreted by immune cells as the target, and the drugs that can directly regulate the function of immune cells in the epidermal immune microenvironment are still in the blank, and the future development prospect is broad. The biological agents are efficacious in the treatment of inflammatory skin conditions, and no single therapy can provide a complete cure for psoriasis. This underscores the ongoing challenges in the pharmacological psoriasis treatment. Furthermore, the extended use of some medications may lead to side effects and contribute to the development of immune tolerance in patients, thereby limiting the long-term efficacy of these drugs. Intervention in the EIME for psoriasis treatment seems a promising area of research. The development of targeted therapeutic strategies and novel intervention drugs could enhance the outcomes for psoriasis patients. Future studies will continue to delve into the mechanisms, safety, and efficacy of these drugs to provide additional therapeutic options and enhance the quality of life for psoriasis patients. In conclusion, an in-depth understanding of these immune cell functions and their correlations with inflammatory pathways can enhance our comprehension of psoriasis pathogenesis and offer valuable insights for future therapeutic approaches. These studies might guide the development of novel therapeutic strategies and improve the early diagnosis and intervention in psoriasis, ultimately enhancing patients’ quality of life.

## Author contributions

LL: Conceptualization, Supervision, Writing – original draft, Writing – review & editing, Data curation. JLu: Conceptualization, Writing – original draft, Data curation. JLi: Conceptualization, Writing – original draft, Investigation. JW: Supervision, Writing – review & editing. XZ: Formal Analysis, Investigation, Writing – review & editing. YM: Supervision, Writing – review & editing. XW: Supervision, Writing – review & editing, Funding acquisition. ZT: Funding acquisition, Resources, Writing – original draft, Writing – review &editing. QZ: Funding acquisition, Resources, Writing – original draft. ZC: Funding acquisition, Resources, Writing – original draft, Writing review & editing.

## Glossary

**Table:**

AS	ankylosing spondylitis
cAMP	cyclic adenosine monophosphate
cIAP1	cellular inhibitor of apoptosis protein 1
Cimzia	Certolizumab polyethylene glycol
CTSG	Cathepsin G
D	dendritic cell
DAMPs	damage-associated molecular patterns
EIME	epithelial immune microenvironment
EMA	European Medicines Agency
FDA	the first Food and Drug Administration
FOXP3	forkhead box transcription factor P3
GM-CSF	granulocyte-macrophage colony-stimulating factor
GPP	generalized pustular psoriasis
HNP1-3	human neutrophil peptide 1-3
ICAM-2	intercellular adhesion molecule-2
IFN-α	interferon-alpha
IKK2	IκBα-kinase-2
ILCs	innate lymphoid cells
JAK	Janus Kinase
JAK-STAT	Janus Kinase-Signal Transducer and Activator of Transcription
KCs	keratinocytes
LC	Langerhans cell
MAPK	Mitogen-Activated Protein Kinase
MDSCs	myeloid-derived suppressor cells
mincle	monocyte-inducible C-type lectin
mTOR	mammalian target of rapamycin
NETs	neutrophil extracellular traps
PAMPs	pathogen-associated molecular patterns
pDCs	plasmacytoid
DCsPDE	phosphodiesterase
PI3K	phosphoinositide 3-kinase
PP	pustular psoriasis
PR3	Proteinase 3
PsA	psoriatic arthritis
PSGL-1	P-selectin glycoprotein ligand-1
RA	rheumatoid arthritis
ROS	reactive oxygen species
TCRs	T cell receptors
Th	T helper
TNFi	tumor necrosis factor inhibitors
TNFR1	TNF receptor 1
TNF-α	tumor necrosis factor-alpha
TRAF2	TNF receptor-associated factor 2
TRAP	TNF associated activation protein
Treg	regulatory T cell
TYK2	tyrosine kinase 2
UVB	ultraviolet B
